# The East Asian crime drop?

**DOI:** 10.1186/s40163-018-0080-x

**Published:** 2018-04-20

**Authors:** Aiden Sidebottom, Tienli Kuo, Takemi Mori, Jessica Li, Graham Farrell

**Affiliations:** 1 Department of Security and Crime Science, UCL Jill Dando Institute, University College London, 35 Tavistock Square, London, WC1H 9EZ UK; 2 Faculty of Human Sciences, Konan Women’s University, 6-2-23, Morikita-machi, Higasinadaku, Kobe, 658-0001 Japan; 3 Department of Applied Social Sciences, The Hong Kong Polytechnic University, Hung Hom, Kowloon, Hong Kong; 4 School of Law, Centre for Criminal Justice Studies, University of Leeds, Leeds, LS2 9JT UK

**Keywords:** Burglary, Car crime, Crime drop, East Asia, Opportunity, Security

## Abstract

The ‘crime drop’ refers to the substantial reductions in crime reported in many industrialised countries over at least the past quarter century. Asian countries are underrepresented in the crime drop literature. Little is therefore known about whether the same type and levels of crime reductions have been observed, and if prevailing explanations hold. In this study, we examine trends in burglary and car crime using police recorded crime data from Hong Kong, Japan and Taiwan. We show that Japan and Taiwan experienced crime drops similar to that reported elsewhere but occurring more recently in the early 2000s. Hong Kong appears anomalous, with a major crime decline emerging from the early 1980s. The study concludes that there is sufficient evidence to justify further research and sets out suggestions to that end.

## Background

The ‘crime drop’ refers to the substantial reductions in crime reported in many western industrialised countries over at least the past quarter century. Most studies refer to a crime drop from the 1990s onwards, but according to the National Crime Victimization Survey, burglary and theft in the United States fell across the 1980s and by 50 and 43% respectively by 1995 (Rand et al. 1997). Subsequently, car theft fell by 78% between 1991 and 2012, violent crime by 70% between 1993 and 2011 (Truman and Planty 2012), and burglary a further 56% over the same period (Walters et al. 2013). What went nationally was also observed at lower levels of geography. In New York City, for example, violent crime fell by 60% and property crime by 64% between 1991 and 2001—the so-called ‘New York Miracle’ (see Zimring 2012). In England and Wales, victim survey data indicate that household crime fell by nearly two thirds between 1993 and 2012, while violent crime more than halved between 1995 and 2012 (ONS 2013). Similar falls in crime have been reported in Canada (Ouimet 2002), Australia (Mayhew 2012), New Zealand (Mayhew 2012) and much of Europe (van Dijk et al. 2012; Tseloni et al. 2010), albeit with some variation in timing and magnitude.^1^

It is a pattern that criminologists failed to predict. Moreover, adequate cross-national accounts of why certain crimes fell where and when they did have proven elusive, what Farrell et al. (2008, p. 17) call “criminology’s dirty little secret”. A recent review of the crime drop literature identified seventeen tested hypotheses (Farrell et al. 2014) ranging from the legalisation of abortion (Levitt 2004) to reductions in the amount of lead in the atmosphere (Nevin 2007). Most of these hypotheses were, however, proposed primarily to explain changes in violent crime in the US, and have subsequently been shown to be inadequate accounts of a) property crime trends in the US, b) crime trends cross-nationally and c) why certain crime types (such as mobile phone theft and various cyber-crimes) have increased in recent years (Farrell et al. 2010). As Tonry (2005, p. 1) observes, “conspicuous by its absence in western criminology is a literature on why crime rates have fallen continuously in some countries and intermittently in others since the early to mid-1990s”.

Farrell and colleagues propose and empirically test an alternative explanation: the ‘security hypothesis’ (Farrell et al. 2011, 2016). Grounded in crime opportunity theory (see Clarke 2012) and building on the work of Clarke and Newman (2006) and van Dijk (2006), this suggests that the falls in crime observed internationally are attributable to reductions in opportunities brought about by growth in and improvements of everyday security. Examples include the increased prevalence of engine immobilisers and central locking systems on vehicles and better door and window locks on households. Three lines of evidence lend support for the security hypothesis. First, it can explain the variation in reductions by crime type across countries and over time, reflecting differences in the extent and quality of relevant security measures. Second, the crime ‘signatures’ expected to follow from the growth in particular security measures are consistent with the predictions of the security hypothesis (Farrell et al. 2016). For example, if car theft has fallen as a result of incremental improvements in vehicle security, then we would expect to see a gradual shift towards older cars accounting for the largest proportion of all cars stolen, since newer vehicles contain better security. The evidence confirms this to be the case (Farrell et al. 2016). Finally, by suggesting that crime follows opportunity, the security hypothesis predicts that growth in crime opportunities in the absence of commensurate security would, all things being equal, produce *increases* in certain crime types. As indicated above, this is indeed the case for mobile phone theft and many cyber-crimes. As Farrell (2013) observes, most of the crime drop hypotheses are *crime generic*, attributing changes in crime to changes in the number or motivation of offenders. For this reason, they are unable to adequately account for the trajectories of *specific* crime types that go against the downward trend.

Farrell et al. (2014) go on to argue that reductions in car crime and burglary as a result of better security might yield wider positive effects. Drawing on research on criminal careers (for example see Svensson 2002), they suggest that blocking opportunities for these crime types effectively removes a pathway through which many offenders first commit crime and then go on to carry out more serious forms of criminality (termed the *debut crime hypothesis*). Viewed this way, improvements in security might likewise produce (lagged) reductions in violent crime.

The crime drop has been described as the “most important criminological phenomenon of modern times” (Farrell et al. 2014, p. 421). Understanding the mechanisms responsible for why certain crimes fell when and where they did promises to yield important lessons on how best to sustain the observed reductions, and replicate them elsewhere. It also offers theoretical insight on the causes of crime. Presently, however, research on the extent, nature and causes of the crime drop is mainly limited to North America, Australasia and Western Europe. Little is known about whether the same type and levels of crime reductions have been observed elsewhere, and whether prevailing explanations, most notably the security hypothesis, hold in other national contexts, including Asia.

There has been some examination of trends in homicide in Asian countries. Homicide is the crime most likely to be recorded consistently across countries, although even here national data can be “subject to under-recording and manipulation” (del Frate and Mugellini 2012, p. 135) and there is cross-national variation in its definition. Using homicide data collated by the United Nations Survey on Crime Trends and Operations of Criminal Justice Systems, del Frate and Mugellini observe that:

*“Among Asian countries, a long-term decrease was observed in 17 out of 24 countries [from 1995 to 2010]. This was the case in particular with Eastern and Southeast Asia: Japan, Singapore, Bhutan, China, Myanmar, Cambodia, India, Taiwan, Thailand and the Philippines”*

(del Frate and Mugellini 2012, p. 140).

Johnson (2008) examines what caused the 70% reduction in homicides in Japan over the last 50 years. He reports that, “more is known about *who* is responsible for the decline than about *what* has caused it. The search for a more satisfying explanation of Japan’s homicide drop should be a high priority in homicide studies” (2008, p. 146). With respect to Southwest Asia, a steep decline in homicide has also been observed for India since 1992 (Ansari et al. 2015). However, such honourable exceptions aside, in the major edited collections and key publications relating to the international crime drop, Asian countries receive little attention. While the present study seeks to begin to bridge this gap in knowledge, to anticipate one of our main conclusions, there is at minimum a need to more systematically review the evidence relating to Asian crime trends. We hope this study provides a catalyst to do so.

The focus of this study is Hong Kong, Japan and Taiwan. It is not possible to generalise from these countries to the whole of East Asia: the study areas are not representative on several key characteristics. However, we use the broad term East Asia in the expectation that there is some benefit from recognising the geographical proximity of these countries but also, thereby, highlighting the lack of crime drop research relating to the region.

## Methods

All of the data used herein are crimes recorded by the police. We recognise the limitations of these data (see van Dijk 2008). Victimization surveys produce more reliable trends as they are less susceptible to manipulation and changes in police recording practices over time. Nevertheless, in view of the scarcity of research on whether a crime drop has occurred in Asia, we contend that the present analysis has the potential to shed new light on the scope of the international crime drop.

Burglary and motor vehicle theft are the focus here because some data on these crime types could be obtained for each country, albeit for different time periods: 1970–2016 for Japan, 1984–2016 for Taiwan and 1978–2016 for Hong Kong. These crime types were also selected because international comparisons reveal that burglary and motor vehicle theft are among the most reliable types of police recorded crime data (van Dijk 2008, p. 21). They are typically more likely to be reported to the police than many personal crimes (often for insurance purposes) although as with any police data they must be used and interpreted with caution. Unlike Japan and Taiwan, our analysis of vehicle crime in Hong Kong relates to theft *from* motor vehicles rather than theft *of* motor vehicles. This is owing to variation in how this crime type is defined. Theft of motor vehicles is not a police crime category in Hong Kong. Instead, such offences may be recorded as either ‘missing motor vehicles’ or ‘taking conveyance without authority’, which includes a broader range of behaviours than that which is covered by the definition of theft of motor vehicles used elsewhere (such as using a vehicle without the consent of the owner). It should be noted, however, that in other settings there is some evidence that ‘theft of’ and ‘theft from’ vehicles tend to track each other over time, even though their rates differ (see e.g. Farrell 2016).^2^

For consistency, annual population data for each country were downloaded from the United Nations Population Division.^3^ These figures acted as the denominator in this study, with crime rates being calculated per 100,000 population for the corresponding year. In what follows, for simplicity we refer solely to ‘the rate’ of crime per 100,000 persons without reference to it being based on police recorded crime data.

## Results

**Fig. 1:**
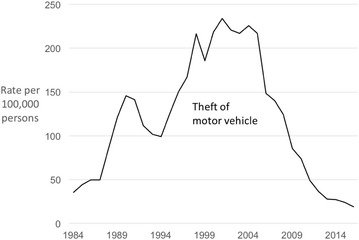
Police recorded theft of motor vehicle in Taiwan 1984–2016 (rates per 100,000 population)

We begin with motor vehicle theft. Following at least two decades of steady increases, the rate of theft of motor vehicles in Taiwan peaked in 2001 then went into prolonged decline, bar the isolated uptick in 2003/2004 (Fig. 1). Between 2001 and 2016, theft of motor vehicles in Taiwan declined by 92%.

**Fig. 2:**
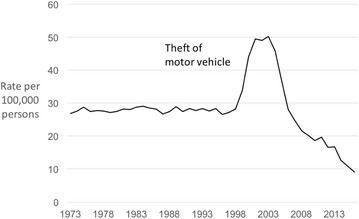
Police recorded theft of motor vehicle in Japan 1973–2016 (rates per 100,000 population)

Japan exhibits a different trajectory (Fig. 2). Between 1973 and 1998, the rate of motor vehicle theft remained largely stable. This is followed by a sharp increase in recorded thefts. We suspect this sudden jump is most probably a police recording artefact; a similar bump is also observed for burglary in Japan over the same time period (Fig. 4). However, if we dismiss the hump in Fig. 2 and focus on theft of motor vehicles from the decade starting 2006, when the pre-existing trend is resumed, it can be seen that theft rates show a substantive and continued fall. More specifically, between 2006 and 2016, theft of motor vehicles in Japan declined by 68%.

**Fig. 3:**
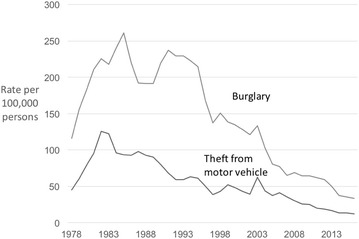
Police recorded burglary and theft from motor vehicle in Hong Kong 1978–2016 (rates per 100,000 population)

The rate of theft from vehicles in Hong Kong increased rapidly in the late 1970s and early 1980s (Fig. 3). However, notwithstanding an uptick in 2003, recorded theft from vehicles in Hong Kong declined steadily for three decades between 1982 and 2016, and by 86%.

The observed falls in motor vehicle theft in Japan and Taiwan are consistent with what is elsewhere referred to as the crime drop. Although the rate of theft *from* vehicles in Hong Kong cannot be directly compared, the data analysed here suggest it has been in decline for considerably longer.

**Fig. 4:**
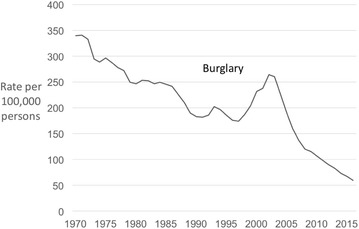
Police recorded burglary in Japan 1970–2016 (rates per 100,000 population)

Turning to burglary, since 1970 burglary has been mostly in long-term decline in Japan (Fig. 4). Between 1970 and 1996, the rate declined steadily and by 48%. An upswing lasting 6 years saw it increase by half (51%) by 2002, albeit from the lower base rate. This was followed by the period of steepest decline such that between 2002 and 2016 the burglary rate in Japan fell 77%. Overall between 1970 and 2016, the burglary rate in Japan reduced by 79%.

**Fig. 5:**
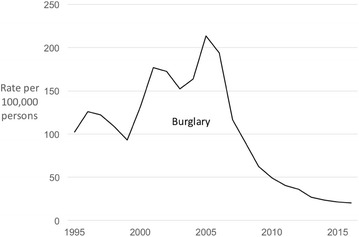
Police recorded burglary in Taiwan 1995–2016 (rates per 100,000 population)

For Taiwan, burglary data were available for 1995–2016 (Fig. 5). The trend in the burglary rate in Taiwan is similar to that of theft of motor vehicles, albeit peaking slightly later. Burglary shows a steady upward trajectory through the 1990s and early 1900s, and peaked in 2005. This marked the beginning of a sharp and sustained decline in burglary. Between 2005 and 2016, burglary in Taiwan fell by 90%. In Hong Kong, burglary rose sharply in the 1970s and early 1980s, peaked in 1985 and has since declined for three decades (again, notwithstanding an uptick in 2003). Between 1985 and 2016, the recorded burglary rate in Hong Kong declined by 83% (Fig. 3).

## Discussion

The ‘crime drop’ refers to the unprecedented reductions in crime reported in many industrialised countries over the past three decades. Research on the extent, nature and causes of the crime drop is, however, mainly limited to North America, Australasia and Western Europe. It is unclear whether similar reductions in crime have been observed elsewhere and, if so, why. The purpose of the present study was to explore whether East Asia has also experienced a ‘crime drop’, focussing specifically on police recorded burglary and car crime in Hong Kong, Japan and Taiwan.

The long-term trend in motor vehicle theft in Taiwan and the more recent trend in Japan is consistent with that found in other high-income countries, most notably Australia, where vehicle theft peaked in 2001 then went into prolonged decline (Farrell et al. 2011). There is strong evidence that the decline in vehicle theft in Australia and elsewhere was due to improved vehicle security, particularly the spread of electronic vehicle immobilizers (Kriven and Zeirsch 2007; Farrell et al. 2011). Vehicle thefts are usually reported to the police and so we are more confident that the trend identified in Japan and Taiwan is not simply a product of changes in, say, police activity and/or recording practices. It is a trend which we feel offers significant potential for further research. Such research might seek to identify data on vehicle security to determine whether improvements over time correspond with the observed reductions in vehicle theft, as would be predicted by the security hypothesis. This would likely involve researchers working in collaboration with the police, the insurance industry, and vehicle and security component manufacturers.

The 30-year decline in theft from vehicles in Hong Kong is somewhat anomalous in the context of previous crime drop research. That is, the onset of the decline appears earlier than has been observed elsewhere. This may offer a challenge to the security hypothesis as presently conceived. Perhaps part of the explanation for the trend lies in Hong Kong’s recent political history, it having been a British colony before returning to China in 1997 (although we can offer no more specific suggestion of a mechanism to reduce theft from vehicles). It is also conceivable that improved vehicle security was introduced earlier in Hong Kong. Further research is needed.

Further research into car theft might also consider the nature and composition of vehicle markets. For instance, a study of young car thieves in the UK conducted around 1990 suggests that Japanese vehicles may have been more secure than others at that time, one offender reporting:

I just look for cars that are easy to nick [steal] – cars that aren’t alarmed, general stuff like Fords and MGs and Austins that are easy to get into. *Nissans, Toyotas* – *mainly Japanese makes like Subaru are really hard to get into because they’ve got awkward locks*.

(Young offender quoted in Light et al. 1993, pp. 48–49, emphasis added).

Crime drop research to date has not, to our knowledge, addressed whether country of manufacture rather than legislation within destination country is a significant determinant of vehicle security levels. Further research is needed. With respect to the present study, however, the initial goal of further research should be to establish whether the trends are real or artefacts of the police recorded crime data.

The decline in burglary in Taiwan and Japan (aside from the 6-year upswing) is similar to that which was discussed in the Introduction as having occurred in the US. It has been suggested that improved household security was a key driver in the falls in burglary in the US (Farrell et al. 2014). There is also strong evidence that improved household security caused the decline in household burglary in England and Wales (Tseloni et al. 2017), and that improved security spread along with double-paned windows (see Farrell et al. 2014; Tilley et al. 2015). While further exploration of the validity of the trends in Taiwan and Japan is warranted, there may also be scope to assess whether similar causal mechanisms underlay the observed reductions in burglary. To this end, it is important to note that the same causal mechanism might be activated by *different* security measures, reflecting variations in context. For example, the spread of double-paned windows in England and Wales is thought to have reduced opportunities for domestic burglary through, amongst other things, *increasing the effort* required for an offender to illegally gain entry to a household. In Japan and Taiwan, it is possible that the observed reductions in burglary might also be attributed to increases in offender effort, but that the *increase*-*effort mechanism* was activated by one or more *different* changes in or improvements to household security, such as increases in private security guards, sensor lights and so on. Focussing on Japan in particular, we conjecture that the uptick in burglary from the late 1990s reflects the increased attractiveness of mobile phones as a target for theft, which caused the increases in burglary as well as a sharp upswing in larceny that we identified in the recorded crime data but which is not shown here.

The three decade-long decline in the rate of burglary in Hong Kong would also benefit from further validation. We note that the trends in burglary and theft from vehicles in Hong Kong are very similar in terms of their overall trajectories. While the prolonged decline in burglary is not dissimilar to that which has been identified for the US, the trend in theft from vehicles appears anomalous. Further research is needed to determine whether improvements in household security can account for the decline in burglary in Hong Kong.

## Conclusion

The contribution of this study is to begin to address the question of whether there has been a crime drop in East Asia comparable to that identified in Europe, North America and Australasia. It offers avowedly simple (descriptive) analyses of long-term trends in car crime and burglary in Hong Kong, Japan and Taiwan. Although the police data used herein must be viewed with caution, if the trends observed are reliable then the study has identified significant grounds for further research in each of the three jurisdictions examined.

We conclude that there is preliminary evidence that a crime drop occurred in Japan and Taiwan for those crime types studied. This conclusion is based on the fact that police data for vehicle theft and burglary are generally reliable and that the timing and trajectory of the crime drop is similar to that identified in other high-income countries. We contend that further research is warranted both on the trajectories of other types of crime and the possible causes of the declines identified in motor vehicle theft and burglary.

The trend observed here in relation to theft of motor vehicles in Hong Kong may question the validity of the security hypothesis in that context. The security hypothesis has become one of the more prominent explanations for why crime has declined internationally. Yet if vehicle crime in Hong Kong fell before improved vehicle security then an alternate explanation must be sought. The use of police recorded crime as the measure here suggests that further exploration of any changes to definitions, and to reporting and recording practices would be a sensible starting point for further study.

## Footnotes

1 It has been suggested that Switzerland is an exception (Killias and Lanfranconi 2012) although others interpret the crime drop as having stalled in that country (van Dijk and Tseloni 2012).

2 It is noteworthy that both ‘taking conveyance without authority’ and ‘missing motor vehicles’ have experienced substantial reductions in Hong Kong in recent decades. Police recorded data for taking conveyance without authority were obtained for 1978 to 2008. Over this period, taking conveyance without authority peaked in 1992 and subsequently fell by 81% by 2008. Police recorded data for ‘missing motor vehicles’ was only available from 2001, whereafter motor vehicle theft steadily declined, and so the prior trend is unknown. Between 2001 and 2016, ‘missing motor vehicles’ in Hong Kong fell by 83%.

3 http://www.un.org/en/development/desa/population/theme/trends/index.shtml.
